# Primary Angioplasty in a Catastrophic Presentation: Acute Left Main Coronary Total Occlusion—The ATOLMA Registry

**DOI:** 10.1155/2020/5246504

**Published:** 2020-07-27

**Authors:** A. Gutiérrez-Barrios, L. Gheorghe, S. Camacho-Freire, F. Valencia-Serrano, D. Cañadas-Pruaño, G. Calle-Pérez, I. Alarcón de la Lastra, E. Silva, D. García-Molinero, A. Agarrado-Luna, R. Zayas-Ruedas, R. Vázquez-García, A. Serra

**Affiliations:** ^1^Departamento de Cardiología Hospital Universitario Puerta del Mar, Cádiz, Spain; ^2^Instituto de Investigación e Innovación en Ciencias Biomédicas de Cádiz, INiBICA, Cádiz, Spain; ^3^Departamento de Cardiología Hospital Juan Ramón Jiménez, Huelva, Spain; ^4^Departamento de Cardiología Hospital Torrecárdenas, Almería, Spain; ^5^Departamento de Cardiología Hospital de Jerez, Cádiz, Spain; ^6^Departamento de Cardiología Hospital de la Santa Creu i Sant Pau, Barcelona, Spain

## Abstract

**Objectives:**

To determine the outcome predictors of in-hospital mortality in acute total occlusion of the left main coronary artery (ATOLMA) patients referred to emergent angioplasty and to describe the clinical presentation and the long-term outcome of these patients.

**Background:**

ATOLMA is an uncommon angiographic finding that usually leads to a catastrophic presentation. Limited and inconsistent data have been previously reported regarding true ATOLMA, yet comprehensive knowledge remains scarce.

**Methods:**

This is a multicenter retrospective cohort that includes patients presenting with myocardial infarction due to a confirmed ATOLMA who underwent emergency percutaneous coronary intervention (PCI).

**Results:**

In the period of the study, 7930 emergent PCI were performed in the five participating centers, and 46 of them had a true ATOLMA (0.58%). At admission, cardiogenic shock was present in 89% of patients, and cardiopulmonary resuscitation was required in 67.4%. All the patients had right dominance. Angiographic success was achieved in 80.4% of the procedures, 13 patients (28.2%) died during the catheterization, and the in-hospital mortality rate was 58.6% (27/46). At one-year and at the final follow-up, 18 patients (39%) were alive, including four cases successfully transplanted. Multivariate analysis showed that postprocedural TIMI flow was the only independent predictor of in-hospital mortality (OR 0.23, (95% CI 0.1–0.36), *p* < 0.001).

**Conclusions:**

Our study confirms that the clinical presentation of ATOLMA is catastrophic, presenting a high in-hospital mortality rate; nevertheless, primary angioplasty in this setting is feasible. Postprocedural TIMI flow resulted as the only independent predictor of in-hospital mortality. In-hospital survivors presented an encouraging outcome. ATOLMA and left dominance could be incompatible with life.

## 1. Background

Patients with myocardial infarction (MI) caused by left main coronary artery (LMCA) represent a high-risk group of patients, and treatment of such lesions is an issue of debate. Contemporary randomized controlled trials (RCTs) have shown that both coronary artery bypass grafting surgery and percutaneous revascularization (PCI) may be considered [1, 2]; nevertheless, the availability of PCI offers a reasonable therapeutic option in patients who are too critically ill to tolerate surgery [3].

Prior RCTs have never included subjects with the most critical ofcoronary pathology circumstances: acute total occlusion of the left main coronary artery (ATOLMA). ATOLMA is a quite uncommon angiographic finding that usually leads to a catastrophic presentation. This entity may be susceptible to associated cardiogenic shock (CS), malignant ventricular arrhythmias, and sudden death unless there are substantial preexisting intercollaterals and complete reperfusion is rapidly established [3–5].

Only limited and inconsistent data have been reported regarding to percutaneous treatment of true ATOLMA. Previously reported studies have been largely confined to small cohorts and also included subtotal occlusion or critical stenosis of the LMCA [6–11].

This study aims to determine the outcome predictors of in-hospital mortality in true ATOLMA (100% occlusion) and to describe clinical presentation and long-term prognosis of these patients.

## 2. Methods

### 2.1. Study Population and Procedure

This is a multicenter retrospective cohort study. The inclusion criteria included patients presenting with ST-elevation MI with the culprit being the unprotected LMCA with a total angiographic occlusion (100%) who underwent emergency primary PCI.

Patients with an LMCA subtotal occlusion, previous patent coronary artery bypass grafting, or iatrogenic acute ATOLMA were excluded.

An independent investigator blinded to all data, except for the coronary angiograms, reviewed all angiograms. The study was carried out in accordance with the principles of the Declaration of Helsinki. The study protocol was approved by the ethics committee.

Angiographic success was defined as a residual stenosis of <30% with a thrombolysis in myocardial infarction (TIMI) flow ≥2.

Two experienced interventional cardiologists retrospectively evaluated the angiographies in a blinded manner to classify collateral circulation (CC) into grades. Disagreement between these assessments was resolved by a third interventional cardiologist. The CC was graded according to Rentrop's classification: grade 0, no filling of the occluded vessel; grade 1, filling of the side branches; grade 2, partial filling of the epicardial vessel; grade 3, complete filling of the epicardial vessel by collateral [12].

To complete follow-up and to determine the clinical events and vital status of the patients, electronic medical records, scheduled visits, and telephone interviews were used.

The primary aim of the study was to determine the significant predictors of in-hospital total mortality. The secondary aims were to describe the incidence and clinical presentation of these patients and to evaluate long-term mortality and major adverse events (MACE) encompassing all-cause mortality, cardiac transplant, new-onset MI, target lesion revascularization (TLR), and definite or probable stent thrombosis (ST) according to the ARC criteria [13].

### 2.2. Statistical Methods

Data were expressed as mean ± standard deviation for continuous variables and compared using the unpaired *t*-test. Categorical variables were expressed as numbers or percentages and compared using chi-square analysis or Fisher's exact test.

A multiple logistic regression analysis was performed to identify independent variables associated with in-hospital mortality.

Variables related to the dependent variable in univariate analysis (*p* < 0.05) were included in the multivariate models.

Survival curves were generated by the Kaplan–Meier method, and differences between groups were analyzed with the log-rank test; a landmark analysis was performed with a landmark of 30 days among patients who were survivors or MACE-free at this time. A *p* value < 0.05 was considered statistically significant. All statistical analyses were performed using SPSS version 22.0 (SPSS Inc., Chicago, IL).

## 3. Results

### 3.1. Incidence and Presentation

Since Jan 2005, in two of the five participating centers, and since Jan 2011, in the other three centers, to Dec 2019, primary angioplasty was performed in 7930 patients; 131 (1.6%) of those were caused by acute unprotected LMCA disease and 46 of those patients had a true ATOLMA and were included in the present study (0.58%).

Clinical characteristics of the overall study population are shown in Table 1. The prevalence of the main cardiovascular risk factors and the presence of a prior history of ischemic cardiopathy were found to be relatively low. The predominant symptom at presentation was chest pain in 47.8%. Forty-one patients (89%) developed CS, only one patient presented in Killip class II (2.1%), and the rest of the patients (4/46) had a Killip score 3. Electrocardiograms at presentation were available for only 18 patients. Anterolateral ST elevation was present in 15 patients (83%), ST-segment elevation in lead aVR in 10 patients (55%), left bundle branch block (LBBB) in three cases (17%), and lateral ST-segment depressions showing signs of extensive transmural ischemia in one patient (5.5%).

Mechanical support devices were performed in patients with a very poor haemodynamic condition; all of them presented Killip class IV (21/21, 100%), and the mean systolic pressure was 61 ± 10 mmHg. Intra-aortic balloon pump and extracorporeal membrane oxygenation (ECMO) were used in 19 (41.3%) and 3 (6.5%) cases, respectively. ECMO was successfully used as a bridge for transplantation in two patients.

37% (17/23) of the patients in which CC was assessed before performing PCI had a Rentrop score of 0, 13% (3/23) had a Rentrop 1, one patient had a Rentrop 2, and the patient who presented a Killip score of two had a Rentrop 3.

### 3.2. Procedural Data

Treatment and procedural characteristics are shown in Table 2. Most of the procedures were performed by the femoral approach (29/46, 63%) and using a 6-French guiding catheter (37/46, 80.5%). Intracoronary imaging was performed in order to optimize the final result in five cases (11%): intravascular ultrasound in four cases and optical coherence tomography in one (Table 2).

**Figure 1:**
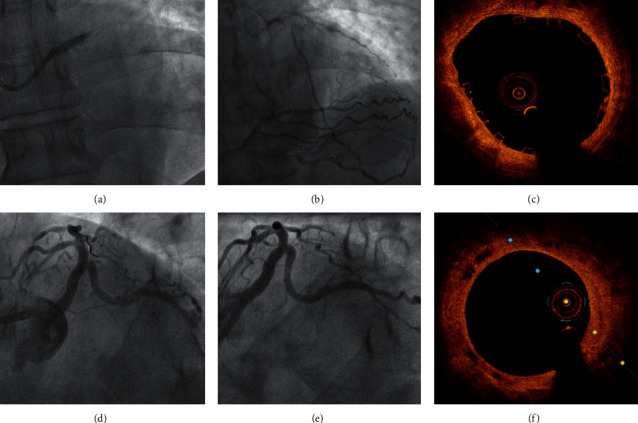
(a) The angiography showed an acute thrombotic occlusion of the unprotected left main coronary artery (ATOLMA). (b) A dominant right coronary artery that contributed Rentrop 3 collateral circulation. (c) The OCT revealed a correct apposition of the bioresorbable vascular scaffold (Absorb 3.5 × 28 mm). (d) Final angiographic result. (e) At 40-month follow-up, an excellent result is maintained in the angiography. (f) The OCT showed reabsorption in process with partial disappearance of the black boxes.

PCI with stent implantation was performed in 69.6% (32/46) of the procedures. Most of the cases received a drug-eluting stent (DES) (25/32, 78%). Exceptionally, in a patient with Rentrop 3, a bioresorbable stent was successfully implanted (Figure 1). Twenty-two patients presented a multivessel disease, and multivessel PCI was performed in 12 of them (12/22, 58.3%).

### 3.3. Mortality and Major Cardiovascular Events

Thirteen patients (28.2%) died in the catheterization laboratory during the procedure, and 7 (15.2%) died in the following 24 hours due to pump failure. Seven patients (15.2%) died during hospitalization, between the second and the twenty-sixth day due to CS (5/7, 71%) and nosocomial sepsis (2/7, 28.5%). The in-hospital mortality rate was 58.6% (27/46). Two patients with INTERMACS profile 3 dependent on ECMO had an emergency transplant due to refractory CS on the 14^th^ and on the 30^th^ day, respectively.

Mean follow-up, among the survivors, was 31 months (interquartile range, 1.4–67 month). One patient, in which a BMS was successfully implanted, died suddenly on day 19 after discharge. Therefore, 16 patients (34.7%) survived without transplantation at 30-day follow-up.

Among the survivors, three patients were admitted to the hospital due to heart failure (HF) on the 21^st^ day and on the second month after discharge. Two patients, with refractory HF, were transplanted in the second and the sixth month. Another patient with a BMS restenosis was successfully treated with a new DES, seven months after discharge. No other patient died during the first-year follow-up.

Therefore, at one-year follow-up, 18 patients (39%) were still alive, including the four cases successfully transplanted, and only 11 patients (24%) were alive and free of MACE or admission for HF. At final follow-up, 10 (21.7%) patients were alive and free of any event (Table 3). The patient treated with a bioresorbable stent was asymptomatic and free of event at 40-month follow-up.

Univariate predictors of in-hospital mortality are reflected in Tables 1 and 2. Multivariate analysis showed that postprocedural TIMI flow was the only independent predictor of in-hospital mortality (Table 4).

CC was not a significant univariate predictor of mortality; nonetheless, CS (100% vs. 50%, *p*=0.01) and one-year MACE were significantly higher in patients with Rentrop 0 at the initial angiography (81% vs. 19%, *p*=0.01).

**Figure 2:**
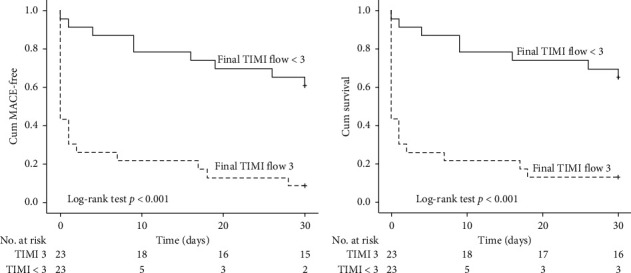
Kaplan–Meier curves showing that the final TIMI flow 3 was significantly associated with 30-day death and 30-day MACE (log-rank test *p* ≤ 0.01 for both).

Kaplan–Meier MACE-free and survival curves at 30-day follow-up showed that both events were significantly lower in patients with a postprocedural TIMI flow 3 compared to those with a final TIMI flow ≤3 (Figure 2).

Data for dual antiplatelet therapy (DAPT) were missing in 11 patients; 51% (18/35) of the patients were treated with ticagrelor, 40% with clopidogrel (14/27), and the rest with prasugrel (3/35, 9%)

## 4. Discussion

Emergency presentation with occlusion of the LMCA is a dramatic and catastrophic coronary event. Except the descriptive cohort reported by Edes et al. [14], the rest of the existing literature includes patients with severe stenosis or subtotal occlusion of the LMCA. As far as we concern, this is the largest reported cohort including exclusively patients with a true ATOLMA (TIMI flow 0) referred to primary angioplasty.

### 4.1. Incidence

Previous studies have reported that the incidence of ST-elevation myocardial infarction (STEMI) caused by LMCA ranged from 0.8 to 2.5% in patients undergoing cardiac catheterization [3, 6, 11, 15–17].

In the present study, a lower incidence was found (0.58%). The discrepancy is related to the fact that previous studies also included subtotal occlusion or critical stenosis of the LMCA, while in this cohort, exclusively, patients with a true ATOLMA referred to primary angioplasty were recruited [4, 6–8, 10, 11, 16].

However, the true incidence of ATOLMA may be underestimated because most of the patients in this clinical setting died before angiography can be performed.

### 4.2. Clinical Presentation

ATOLMA usually results in severe ventricular dysfunction leading to a rapid haemodynamic deterioration and a catastrophic clinical presentation [4, 6].

Previously reported studies showed an incidence of CS ranged between 62 and 83% [5–7, 11, 16–19] and of mechanical ventilatory support requirement (invasive or noninvasive) between 23 and 89% [6, 11, 16].

Our study, in agreement with that reported by Edes et al., including strictly true ATOLMA, reflects even a poorer clinical presentation: 89% (41/46) of the patients developed CS [14]; 67.4% (31/46) required orotracheal intubation and invasive ventilatory support, and cardiopulmonary resuscitation maneuvers were necessary in 67.4% (31/46) patients.

All except one patient presented Killip class III-IV, which reflect the poor clinical status of these patients at presentation. ATOLMA should be suspected in patients with STEMI accompanied by these potentially devastating presentations, and an early invasive strategy should be encouraged in these patients [3, 6, 8].

### 4.3. Prognosis

The “LMCA shock syndrome” originally described by Quigley et al. in 1993 showed that when STEMI occurs with CS and severe LMCA stenosis, prognosis, regardless of management, was extraordinarily poor with a mortality rate of 94% [5, 11, 20, 21].

A more contemporary approach using new-generation stents, haemodynamic support, and new treatments, in particular new antiplatelet therapies, and also the increased experience with treating LMCA percutaneously and PCI in the context of STEMI, have slightly improved results and prognosis. However, STEMI caused by LMCA disease still has a poor prognosis, with the exception of the study by Liu et al., in which a surprisingly low in-hospital mortality rate (5.1%) and 30-day mortality (6.2%) were found [8]. Most of the studies reported an in-hospital mortality rate of 31–58% and a 30-day mortality of 36–63% [3, 6, 7, 10, 11, 16–18, 22–25].

Little evidence exists regarding the mortality rate in true ATOLMA (TIMI 0). However, as expected, the limited available information shows a poorer prognosis in this scenario when compared with critical or subtotal LMCA occlusion. De Luca et al. reported a mortality rate of 60% in the subgroup of patients with a TIMI flow 0, very similar to that reported by Edes et al. (56%) and Yip et al. (62%, 5/8) in those subgroup of patients.

Two more contemporary studies revealed a similar outcome: YAP et al. found a mortality rate of 54% (22/41) and Homorodean et al. 63% (12/19), despite these results referring to TIMI 0-1 patients and not exclusively to TIMI 0 cases [6, 10, 11, 14, 18].

Concordantly with previous observations, in the present study, although restoration of the coronary flow is mostly successful (80.4%), a rather significant in-hospital mortality was observed (58.6%, 27/46). But, even the true mortality of ATOLMA may be underestimated as many patients could not be taken to the catheterization laboratory before dying.

In this cohort, patients surviving the initial hospitalization showed an encouraging prognosis; only one patient died during follow-up (6%, 1/19), which is comparable to the findings previously reported with a survival rate of 83–90% among in-hospital survivors [3, 5, 6, 11, 14, 17, 22, 25].

### 4.4. Predictors of Mortality

#### 4.4.1. Collateral Circulation

The role of early recruited CC in STEMI remains controversial. Some previous reports have shown lower mortality and morbidity rates in well-collateralized patients, but others have not [26–29].

Regarding LMCA PCI in the setting of primary angioplasty, it has been suggested that the presence of well-developed collateralization is a crucial predictor of survival [4, 5, 11, 16, 18, 30].

As only true ATOLMA was included in our study, the clinical presentation was often devastating, and the operator decided not to perform a contralateral injection before the LMCA PCI in 23/46. Therefore, CC grade information was missing in 50% of the procedures, and well-developed CC (Rentrop class ≥2) was confirmed only in two cases (8.7%). Nevertheless, the absence of CC (Rentrop 0) was significantly higher in patients with CS at presentation and was related to one-year MACE, which highlights the role that CC may play in ATOLMA patients. Nonetheless, in our study, since the CC grade was assessed only in 50% of the procedures, CC was not a univariate predictor of in-hospital mortality and consequently excluded in the multivariate model. Certainly, the role of CC in ATOLMA remains to be further elucidated.

#### 4.4.2. Right Dominance

In STEMI treated with primary angioplasty, right coronary dominance confers a better prognosis than left dominance, and it is explained by the fact that right dominance has a greater division of vasculature supplying the left ventricle (into 3 “parts”), whereas left dominance means that most of the myocardium is essentially dependent on 2 arteries [31].

Concerning ATOLMA, this vasculature division dependent on coronary dominance becomes even more relevant since LMCA supplies those two arteries. Therefore, it is not surprising that dominance has been related to survival in patients with subtotal LMCA occlusion [11]. In true ATOLMA, it has been suggested that only patients with right dominance will survive to receive a diagnosis and invasive treatment [5, 14].

In agreement with these observations, we found that 100% (46/46) of the patients included in our study had right dominance, probably because left main dominance patients may die before being transferred to cardiac catheterization. Our findings and previous results suggest that most probably ATOLMA and left dominance could be incompatible with life, especially in the absence of well-developed CC [14].

#### 4.4.3. Final TIMI Flow

TIMI flow grades have been shown to have significant prognostic implications among patients undergoing reperfusion therapy for STEMI [32–34], and similar results have been reported in the setting of primary angioplasty in LMCA [6, 10, 16].

Concordantly, our study proved that postprocedural TIMI flow in ATOLMA patients was the only independent predictor of in-hospital mortality in the multivariate model. The TIMI flow achieved was also significantly related to short-term MACE and mortality as reflected in the Kaplan–Meier curve (Figure 2). Therefore, restoration of TIMI 3 flow is crucial in this situation. However, TIMI flow 3 was only achieved in 50% (23/46) of our patients, which is significantly lower than general STEMI where TIMI 3 is obtained in >90% of procedures, or with not-true ATOLMA where it ranges from 66% to 86% [6, 10, 11, 18, 34].

Obtaining a final TIMI 3 flow can be challenging in this catastrophic scenario, but according to our data and previous knowledge reported, every effort must be made to achieve this goal.

## 5. Limitations

This was a retrospective study with a small sample size. Like all observational studies, the present study is prone to biases from its nonrandom assignment of exposures. CC was not assessed before PCI in 50% of the procedures. Furthermore, while this study focused on in-hospital mortality, the unquestionably high prehospital mortality was not quantified. One inherent limitation is that there are differences in the nature and the type of hospital facilities (e.g., intra-aortic balloon pump, ECMO, or in-site transplant availability). The long period of inclusion may have introduced bias due to changes in technology and management.

## 6. Conclusions

Clinical presentation of ATOLMA patients with MI referred to primary angioplasty is usually catastrophic; most patients presented in CS and CPR maneuvers and OTI were frequently required.

Emergency primary PCI provides a feasible treatment option in this context, yet in-hospital mortality remains quite high. Since the final TIMI flow was the only independent predictor of in-hospital mortality, every effort must be made to achieve this goal. Long-term mortality rates for survivors are reasonably encouraging.

Interestingly, all of our patients had right coronary dominance, suggesting that presumably ATOLMA and left dominance could be incompatible with life.

## Figures and Tables

**Table 1:** Clinical and angiographic characteristics.

	Overall (*n* = 46)	In-hospital mortality		
		Yes (*n* = 27)	No (*n* = 19)	*p*
Age, *y* (SD)	62.8 ± 12	65 ± 5	59 ± 4.8	0.1
Male, *n* (%)	36 (78)	20 (74)	16 (84)	0.5
Diabetes, *n* (%)	7 (15.2)	6 (22)	1 (5)	0.2
Hypertension, *n* (%)	16 (34.8)	7 (37)	9(33)	0.8
Dyslipidemia, *n* (%)	12 (26.1)	9 (33)	3 (16)	0.2
Smoking, *n* (%)	23 (50)	12 (44)	11 (58)	0.3
Prior IC (%)	3 (6.5)	3 (11)	0 (0)	0.25

*Presentation (%)*				
Cardiogenic shock	41 (89)	26 (96)	15 (79)	0.08
Heart rate (bpm)^*∗*^	106.5 ± 15	109 ± 14	102 ± 15	0.2
Systolic blood pressure (mmHg)^*∗*^	70.5 ± 20	67 ± 17	75.5 ± 24	0.3
Cardiac arrest at presentation	8 (17.4)	4 (15)	4 (21)	0.5
Ventricular tachyarrhythmia	17 (37)	10 (37)	7 (37)	1
TIMI flow 0 at presentation	100%	27 (100)	19 (100)	1
Multivessel disease	21 (45.7)	11 (41)	11 (58)	0.37
Right coronary disease	9 (19.6)	4 (15)	5 (26)	0.45
Right coronary dominance	46 (100%)	27 (100)	19 (100)	1
Rentrop 0	17/23 (74)	15 (79)	2 (50)	0.2
Symptom to balloon	187 ± 51	208.5 ± 81	158 ± 49	0.3
FMC to balloon	117 ± 52	139.7 ± 90	87.7 ± 19	0.3

^*∗*^Data available from 23 patients. IC: ischemic cardiopathy; OTI: orotracheal intubation; CC: collateral circulation; FMC: first medical contact.

**Table 2:** Treatment and procedural characteristics.

	Overall (*n* = 46)	In-hospital mortality		
		Yes (*n* = 27)	No (*n* = 19)	*p*
Orotracheal intubation	31 (67.4)	23 (85)	7 (37)	0.06
Ventricular assistance device	21 (45.6)	11 (40)	10 (53)	0.4
IABP	20 (43.5)	11 (41)	9 (47.4)	0.65
ECMO	3 (6.5)	0 (0)	3 (15.8)	0.06
CPR	31 (67.4)	21 (78)	10 (53)	0.07
GP IIa/IIIb inhibitors	22 (47.8)	12 (44)	15 (56)	0.2
Vasoactive drugs	41 (89.2)	25 (96)	15 (79)	0.07

*Angioplasty*				
Angiographic success	37 (80.4)	18 (67)	19 (100)	**<0.001**
Radial approach	16 (34.8)	6 (22)	10 (53)	**0.03**
Seven French catheter	8 (17.4)	2 (7)	6 (32)	0.05
LM stent deployed	32 (69.6)	15 (56)	17 (90)	**0.01**
LM bare metal stent	7 (21.8)	2 (13)	5 (30)	0.4
LM stent diameter (mm)	3.5 ± 0.4	3.5 ± 0.5	3.5 ± 0.2	1
Complex LM technique	6 (13)	3 (11)	3 (16)	0.7
LM stent predilation	15/32 (47)	10 (67)	13 (77)	0.7
LM stent postdilation	15/32(47)	7 (47)	8 (47)	1
Thrombus aspiration	19 (41.7)	13 (48)	6 (32)	0.2
Intracoronary imaging	5 (10.9)	1 (4)	4 (21)	0.14
Contrast volume (ml)	177 ± 132	189 ± 161	161 ± 87	0.6
Final TIMI flow	2.2 ± 0.3	1.78 ± 0.4	2.8 ± 0.2	**<0.001**
Final TIMI flow 3	23 (50)	8 (30)	19 (70)	**0.001**

IABP: intra-aortic balloon pump; ECMO: extracorporeal membrane oxygenation; CPR: cardiopulmonary resuscitation; LM: left main.

**Table 3:** Outcomes of the study population.

*In-hospital outcomes, n (%)*	
In-cath lab mortality, *N* (%)	13 (28.2)
In-hospital total mortality	27 (58.6)
Cardiac arrest	31 (67.4)
Cardiac transplant	2 (4.3)
Major bleeding complications	0 (0)

*One-year outcomes, n (%)*	
Total mortality	28 (60.9)
Cardiac transplant	4 (8.7)
TLR	1 (2.2)
Definite or probable ST	1 (2.2)
Non-fatal MI	1 (2.2)
MACE	33 (71.7)
Heart failure admission	4 (8.7)
Non-fatal stroke	0 (0)

*Total follow-up outcome, n (%)*	
Total mortality	28 (60.9)
MACE	34 (73.9)
Heart failure admission	5 (10.8)

**Table 4:** Predictors of in-hospital mortality (multivariate analysis).

	In-hospital mortality	
	HR (95% CI)	*p*
Final TIMI flow	0.23 (0.1–0.36)	**<0.01**
LM stent	0.1 (−0.21 to 4.3)	0.5
Radial approach	0.25 (0.005–0.5)	0.054
Angiographic success	0.12 (−0.5 to 0.8)	0.6

## Data Availability

The SPSS database data used to support the findings of this study are available from the corresponding author upon request.

## Supplementary Materials

Supplementary Materials Two representative example cases of left main acute occlusion have been submitted as the supplementary material. One of them with the TIMI 3 flow result and the other with a TIMI 1 flow result. Both cases were summarized in a one-minute video with titles and subtitles explaining the procedures.
